# Infant feeding practices and parental perceptions during the 2022 United States infant formula shortage crisis

**DOI:** 10.1186/s12887-023-04132-9

**Published:** 2023-06-24

**Authors:** Karina Cernioglo, Jennifer T. Smilowitz

**Affiliations:** 1 Department of Food Science and Technology, University of California Davis, 1139 Robert Mondavi Institute, Davis, CA 95616 USA; 2 Foods for Health Institute, University of California Davis, Davis, CA USA

**Keywords:** Infant formula shortage, Infant feeding practices, Breastfeeding, Health equity, Regulatory policies, Food insecurity, Healthcare policies

## Abstract

**Background:**

In May of 2022, parents living in the United States experienced a dramatic infant formula shortage caused by supply chain issues and the recall of several infant formula products over contamination concerns.

**Methods:**

An anonymous, electronic, cross-sectional survey was designed to understand infant feeding practices, parental experience and perceived support during the crisis.

**Results:**

Ninety-nine parents that lived in the U.S. and fulfilled study criteria completed the survey. 66% of respondents were female, and 75% of respondents were recipients of the Special Supplemental Nutrition Program for Women Infant Children (WIC). Parental mean age was 30.0 years, and the mean infant age was 26.8 weeks. The number of individuals that used at least one unsafe infant feeding practice increased from 8% before the infant formula shortage to 48.5% during the shortage (p < 0.001). 79% of parents fed their infants U.S. infant formula brands and 39% of parents fed their infants imported infant formula brands before the shortage which were significantly reduced during the shortage to 27% (p < 0.005) and 11% (p < 0.005), respectively. The percentage of parents that reported infant feeding practices before and during the infant formula shortage significantly increased from 2 to 28% for banked donor milk use (p < 0.005); 5–26% for use of human milk from informal sharing (p < 0.005); and 2–29% for use of watered-down infant formula (p < 0.005). The resources that parents reported as most helpful in navigating the crisis differed by parental sex and WIC recipient status and included other parents, friends, and family; lactation consultants; healthcare providers; and WIC.

**Conclusions:**

Our study found that feeding practices in response to the infant formula shortage may pose health risks to infants including nutrition and food insecurity. These data suggest the need for policy changes within regulatory and the healthcare system to provide families with clinical prenatal and postnatal lactation support, access to pasteurized banked donor milk, and access to more commercially available products.

**Supplementary Information:**

The online version contains supplementary material available at 10.1186/s12887-023-04132-9.

## Background

In 2022, the United States (U.S.) faced an acute infant formula shortage that magnified a chronic, less severe shortage that occurred over the past several years [1]. As early as 2020, supply chain issues related to the COVID-19 pandemic contributed to nation-wide shortages of several household items, including infant formula [2]. The 2022 shortage was initiated by a recall by Abbott Nutrition in February 2022. Abbott Nutrition, is the largest U.S. infant formula manufacturer and provides 40% of the nation’s infant formula as either a supplement to or a substitute for human milk for millions of babies [3]. The infant formula company voluntarily recalled several brands of its powdered formula products due to bacterial contamination from *Cronobacter sakazakii* [4]. For four months, Abbott Nutrition also voluntarily shut down a manufacturing plant in Michigan that was found to be connected to the contamination; this is also the site of country’s largest infant formula manufacturing plant [1]. This led to a dwindling supply of infant formula that was further exacerbated by restrictive U.S. trade and tariff policies that limit the availability of safe imported products [5]. By the end of May 2022 the shortage reached its peak with a national out-of-stock rate for infant formula as high as 90% in several states [6]. Because millions of U.S. families rely on infant formula [7], the crisis left parents to face uncertainty in safely feeding their infants [8].

The infant formula shortage was especially distressful for low-income families that participate in the Special Supplemental Nutrition Program for Women, Infants, and Children (WIC) [9]. WIC is a federally funded program that provides resources including access to infant formula to low-income pregnant and postpartum women, infants, and children. WIC recipients comprise the majority of infant formula consumers with an estimated consumption of 56% of all U.S. infant formula in 2018 [10]. WIC agencies competitively bid for contracts from three U.S. infant formula manufacturers for the least expensive contract [5] and WIC recipients receive formula from one of these manufacturers designated by their state [11]. In 2022, Abbott Nutrition dominated WIC contracts [3]. Thus, WIC recipients were severely impacted by the infant formula shortage.

Due to the inaccessibility of infant formula during the 2022 shortage, health officials were concerned parents would rely on deleterious feeding practices. During the COVID-19 pandemic, 33% of families who used infant formula reported experiencing challenges in obtaining infant formula because formula was sold out, they had to travel to multiple stores, or formula was too expensive. These families also reported responding to the shortage by using deleterious feeding practices such as diluting formula with extra water or cereal, preparing smaller bottles or saving leftover mixed bottles for later [12]. These deleterious feeding practices increase the health and safety risks of infants who rely on infant formula whether exclusively or as a supplement to human milk. The American Academy of Pediatrics (AAP), Centers for Disease Control and Prevention (CDC) and Food and Drug Administration (FDA) recommend against: (1) modifying formula such as diluting it with water as it can impair growth and development and result in electrolyte and mineral disturbances [13]; (2) making homemade formula due to the high risk of contamination and infections from its preparation and nutrient deficiencies [14]; (3) feeding cow’s milk before one year of age due incomplete digestibility and increased risk for intestinal bleeding and iron-deficiency anemia [15]; and (4) informal human milk sharing due to possible health and safety risks from exposures to medications, illicit drugs, infectious diseases, or alterations [16]. To date, no studies have reported the effects of the 2022 infant formula shortage on infant feeding practices and parents’ experiences and sentiments. The purpose of this prospective study was to investigate infant feedings practices across the United States during the infant formula shortage that may pose nutritional and safety concerns. We compared responses between the individuals most likely to be affected the greatest by the shortage (WIC recipients vs. non-WIC recipients) [17] and between parental sexes [18]. The second goal of this study was to understand parental experiences and sentiments during the crisis to identify areas within regulatory and healthcare policies and programs that could assist in mitigating future infant feeding crisis.

## Methods

### Subjects and design

Parents of infants in the U.S. were recruited using paid targeted advertisements on various social media platforms to participate in an anonymous, cross-sectional, electronic survey. Participants completed the survey between May 25, 2022, and June 7, 2022. Individuals who completed the survey and passed a multi-tier validation process, received a $15 gift card. Parents were eligible to participate if they fulfilled the following criteria: lived in the U.S. during the infant formula shortage; were the parent of an infant who was 12 months old or younger during the infant formula shortage; and experienced challenges in feeding their infant due to the infant formula shortage (used any amount of infant formula to feed their infants). Parents reported their demographics and answered questions about their infant feeding practices, experiences, and sentiments in response to the infant formula shortage. The study was approved by the UC Davis Institutional Review Board (IRB ID: 1,920,147).

The online survey was created in Qualtrics 2022 (Provo, Utah, USA) and included 42 unique questions. Participants answered yes/no, multiple choice, rating on a sliding scale (0–100%), and open-ended questions. The survey contained questions about demographics including, infant and parental age, socioeconomic status, and ethnicity. Participants were asked what their infants typically ate over a 7-day period right before and a 7-day period during the most challenging time during the infant formula shortage. Additionally, use of human milk from informal sharing, homemade infant formula, watered-down formula and expired infant formula were aggregated into one variable as “unsafe infant feeding practices” for a 7-day period right before and a 7-day period during the most challenging time of the shortage. Participants were asked to select all of the resources that provided guidance or support in feeding their infants during the shortage. Participants were also asked to rate on a sliding scale from 0 to 100% how helpful a list of resources *have been* with providing guidance or support to feed their infants or to select “non-applicable” if they did not experience any help from the listed resource. Infant feeding sources and resources used in this survey were adapted from the Infant Feeding Practices Study II (IFPS II) [19] which differentiated lactation consultants from other healthcare providers and included up-to-date online resources such as social media, and blogs.

Participants were asked to rate on a sliding scale from 0 to 100% how helpful a list of activities *would be* in helping families feed their infants in the near future or to select “non-applicable” if they were unsure. These activities were compiled from comments posted on several multiple social media outlets (Reddit, Facebook, Twitter) by parents during the height of the 2022 infant formula shortage. The survey used in this study is available as Additional File 1.

### Data validation

To ensure the data collected from the study survey was reliable, the original data underwent systematic quality control in a blinded fashion and only a subset of the original responses that met all quality control criteria were included for statistical analyses. Quality control measures included removal of surveys from data analysis if they met the following exclusion criteria: incomplete, repeat responders, non-U.S. IP addresses, identical IP addresses associated with different states; duplicate email addresses; inactive emails; containing repetitious patterns; suspicious comments or did not follow the survey instructions. Individuals whose study survey met the above exclusion criteria were emailed a “validation” survey with additional security measures to verify their identity and original responses to the study survey. Validated data from individuals who met all inclusion criteria were included in the final statistical analyses.

### Statistics

All statistical analyses were conducted using IBM SPSS Statistics version 27 and figures were generated in Graphpad PRISM v.9. Statistical significance was considered as p < 0.05. Bonferroni-adjusted p-values for multiple comparisons are reported herein. Descriptive statistics (means, standard deviations, ranges, frequencies, and percentages) are reported for demographic, reproductive history, infant diet, and infant feeding practices and parental experiences, and sentiments.

To understand how infant feeding changed in response to the infant formula crisis, parents were asked what their infants typically ate over a 7-day period right before and a 7-day period during the most challenging time of the infant formula shortage. Data were treated as binary (yes/no) responses and the McNemar test was used to determine if there were differences for each food item before and during the infant formula shortage. Because no one selected “other” or “sweet foods, candy, cookies, cake, etc.”, only twenty-five food items were statistically analyzed.

The survey prompted parents to select all that apply from a list of nine resources that provided guidance or support in feeding their infants during the infant formula shortage. Data were treated as binary (yes/no) responses and Fisher’s exact 2-tailed test was used to determine differences in the number of individuals who received guidance or support from each resource according to WIC status (recipient of WIC benefits or not) and parental sex (female or male).

Parents who selected “yes” to using any of the nine resources, were asked to rate on a scale 0-100% how helpful nine resources *have been* in providing guidance or support in feeding their infants during the infant formula shortage. Parents were also asked to rate on a scale 0-100% how helpful they think fourteen activities *would be* in helping families feed their babies in the near future. Kruskal-Wallis one-way analysis of variance for non-parametric data was used to identify differences in rank scores by WIC status (recipient of WIC benefits or not) and parental sex (female or male).

## Results

A total of 2,043 individuals completed the study survey, of which, 99 completed surveys were verified and fulfilled all inclusion and exclusion criteria for final data analyses. Respondents resided in 21 unique U.S. states and 62% of respondents resided in the state of California (Additional File 2). All survey respondents identified themselves as the biological parent of an infant who consumed some amount of infant formula during the infant formula shortage. At the time of the survey, parental mean age was 30.0 years. 66% of parents were female and 34% of parents were male. 76% of parents were white, 19% of parents were black, 4% of parents were Asian and 1% of parents reported they were unsure of their race. 75% of parents had reported that they or their infant’s co-parent had received benefits from WIC within the past 12 months of completing the survey (Table 1). There was no relationship between parental sex and WIC status.

**Table 1:** Parental Demographics^1^

	Mean	±	SD	Range
Age, Parent (yrs)	30.0	±	3.6	22–41
Parental Sex, % (n)				
Female	65.7% (65)			
Male	34.3% (34)			
Ethnicity, % (n)				
Hispanic	5.1% (5)			
Non-Hispanic	93.9% (93)			
Decline	1.0% (1)			
Race, % (n)				
Asian	4.0% (4)			
Black/African American	19.2% (19)			
White	75.8% (75)			
Unsure	1.0% (1)			
Education, % (n)				
High school	4.0% (4)			
High school graduate	11.1% (11)			
Some college	34.3% (34)			
Associate’s degree	11.1% (11)			
Bachelor’s degree	34.3% (34)			
Master’s degree	3.0% (3)			
Professional or doctorate	2.0% (2)			
Marital Status, % (n)				
Married/unmarried couple	93.9% (93)			
Divorced/separated	3.0% (3)			
Never married	3.0% (3)			
Household Size (people), % (n)				
2	1.0% (1)			
3	44.4% (44)			
4	27.3% (27)			
5	26.3% (26)			
6	1.0% (1)			
Household Income, % (n)				
Less than $25,000	1.0% (1)			
$25,000 to $34,999	1.0% (1)			
$35,000 to $49,999	5.1% (5)			
$50,000 to $74,999	63.6% (63)			
$75,000 to $99,999	13.1% (13)			
Greater than $100,000	16.2% (16)			
Receiving WIC Benefits, % (n)				
Yes	74.7% (74)			
No	25.3% (25)			
Parity^2^, % (n)				
Primiparous	84.6% (55)			
Multiparous	15.4% (10)			

^1^*n* = 99

^2^*n* = 65, females only

85% of female parents were primiparous who had delivered an infant within the past fourteen months since completing the survey. One parent delivered multiple infants at labor and one infant was born to the biological parent by surrogacy. At the time of the survey, infants’ mean age was 26.8 weeks, and most of the infants were at the age prior to extensive complementary feeding (n = 38 infants were 4–6 months of age and n = 36 infants were 7–9 months of age). 94% of infants were born at term (≥ 37 weeks gestation) and 19% of infants required a specialty formula.

### Infant feeding practices before and during the shortage

**Fig. 1:**
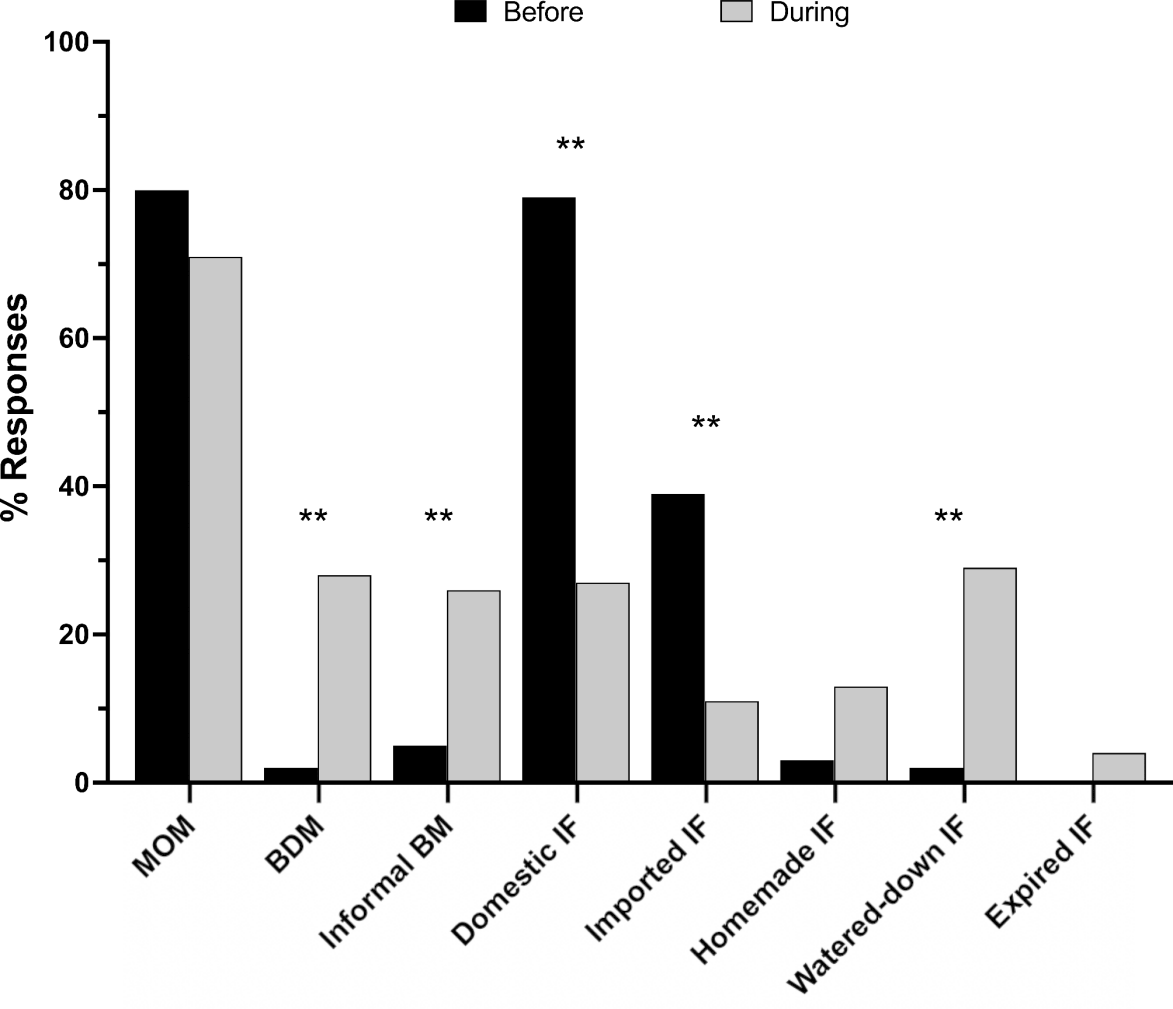
Infant Feeding Practices Before and During the Infant Formula Shortage. MOM = mother’s own milk; BDM = banked donor milk; BM = breast milk; IF = infant formula. **p < 0.005

To understand how infant feeding changed in response to the infant formula crisis, parents were asked to select the foods and liquids their infants typically ate over a 7-day period right before and a 7-day period during the most challenging time of the infant formula shortage. Prior to the shortage, 80% of all infants were fed some amount of their mothers’ own breast milk in combination with formula but this number did not significantly change in response to the infant formula shortage. However, other infant feeding practices significantly changed in response to the crisis. For example, consumers of banked donor milk significantly increased from 2 to 28% (p < 0.005) and consumers of human milk through informal sharing increased from 5 to 26% (p < 0.005). Use of infant formula also significantly changed in response to the crisis. 79% of parents fed their infants U.S. infant formula brands and 39% of parents fed their infants imported infant formula brands before the shortage and these percentages were significantly reduced during the shortage to 27% and 11%, respectively (p < 0.005). Additionally, the use of watered-down infant formula significantly increased from 2% before to 29% during the shortage (p < 0.005) (Fig. 1). In this cohort, users of homemade formula increased from 3 to 13%, but there was insufficient power to detect a significant difference (uncorrected p value < 0.05). There was no significant difference between the number of parents who fed expired infant formula to their infants in response to the infant formula shortage.

The use of cow milk, watered-down cow milk, goat milk, watered-down goat milk, plant-based milks, toddler formula, baby cereal or other food items were not significantly different in response to the infant formula shortage (Additional File 3).

### Parental experiences and sentiments

**Fig. 2:**
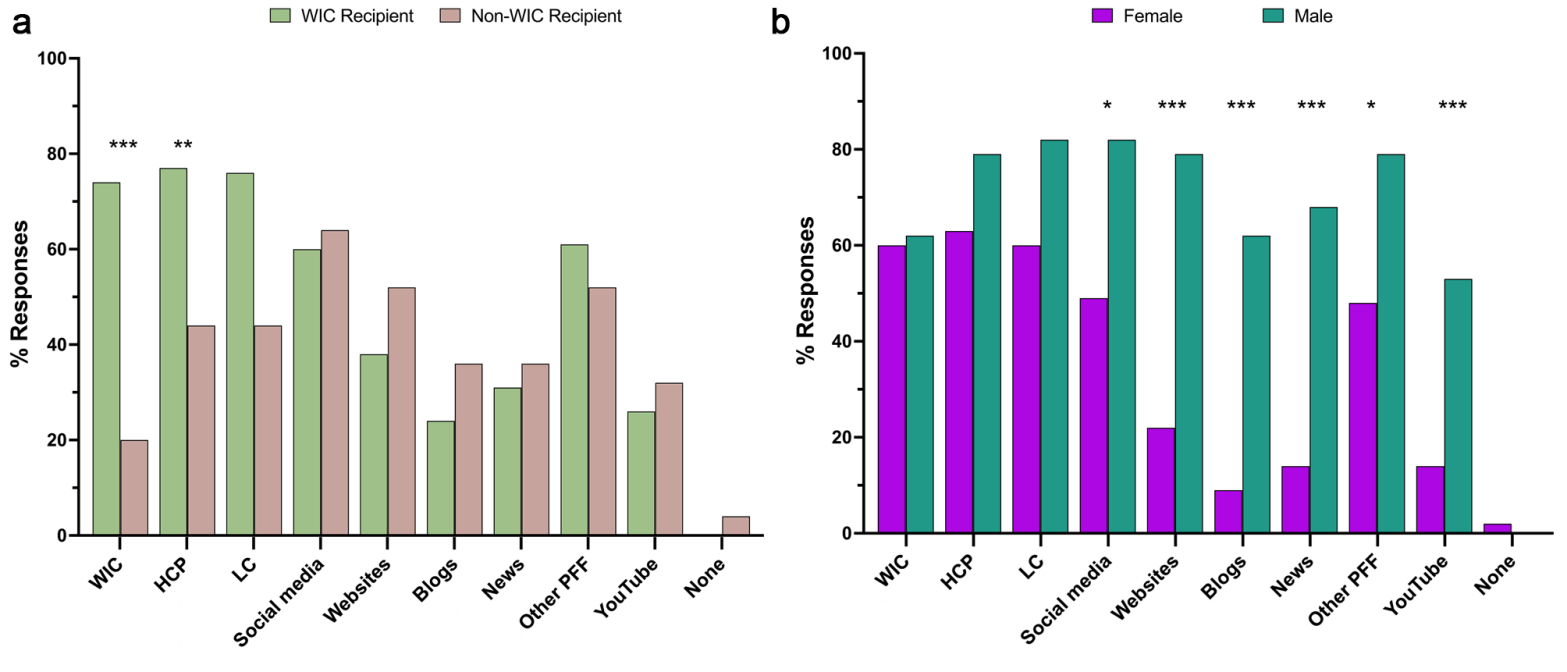
Perceived Guidance and Support for Feeding Infants. (**A**) WIC vs. non-WIC recipients. (**B**) Female vs. Male. WIC = Women, Infants & Children; HCP = healthcare providers; LC = lactation consultants; Other PFF = other parents, friends, family. * p < 0.05; ** p = 0.05; *** p < 0.005

Parents were asked to select the resources that provided the most guidance or support to help them feed their infants during the shortage. More than 50% of parents selected that the following resources provided them with guidance or support in feeding their infants during the infant formula shortage: WIC, healthcare providers, lactation consultants, and other parents, friends and family. More WIC recipients reported that WIC (p < 0.005), healthcare providers (p < 0.05) and lactation consultants (p = 0.05) provided guidance or support, as compared with non-WIC recipients (Fig. 2A). More male parents reported that social media (p < 0.05), websites by health authorities (p < 0.005), blogs (p < 0.005), news (p < 0.005), other parents, friends, and family (p < 0.05), and YouTube videos (p < 0.005) provided guidance or support compared with females (Fig. 2B). There was no relationship between WIC status and parental sex.

**Fig. 3:**
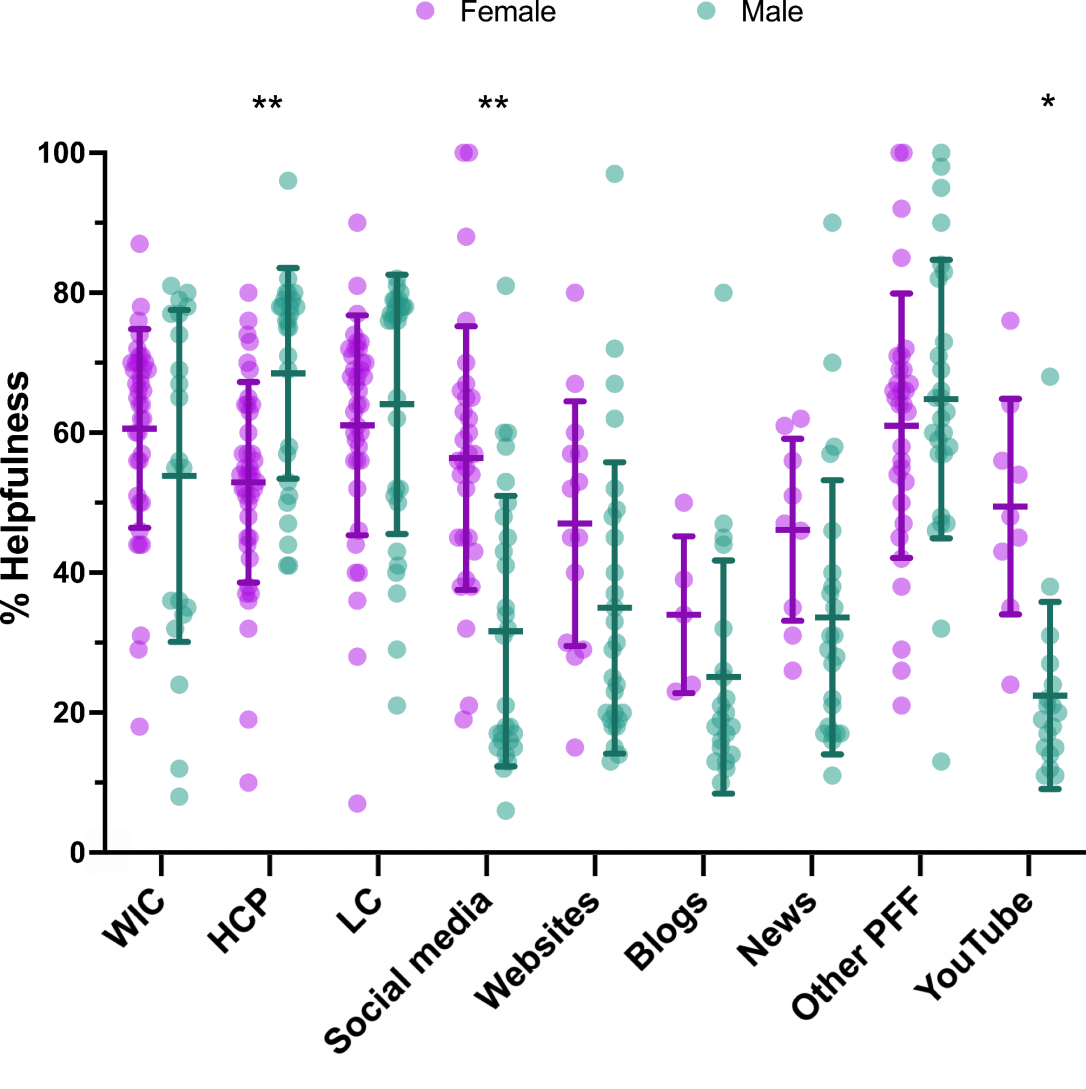
Percent Helpfulness Scores on Resources for Feeding Infants. WIC = Women, Infants & Children (n = 60); HCP = healthcare providers (n = 68); LC = lactation consultants (n = 66); social media (n = 59); websites (n = 41); blogs (n = 26); news (n = 32); other PFF = other parents, friends, family; YouTube (n = 27). * p < 0.001; ** p = 0.0001

Parents were asked to rate on a scale from 0 to 100% how helpful a list of resources *have been* in providing guidance or support to feed their infants during the infant formula shortage or to select “non-applicable” if they did not experience any help from the resource listed. Amongst all participants, the following resources were rated a mean score above 50% for helpfulness: WIC, healthcare providers, lactation consultations, and other parents, friends and family. There were no differences in ratings for the listed resources between WIC recipients vs. non-WIC recipients. Male parents reported higher mean scores on helpfulness from healthcare providers compared with female parents (68.5% vs. 52.9%, p < 0.0001). On the other hand, female parents reported higher mean scores on helpfulness from social media (56.4% vs. 31.6%, p < 0.0001) and YouTube (49.4% vs. 22.4%, p < 0.001) compared with male parents (Fig. 3).

Parents were asked to rate on a scale from 0 to 100% how helpful a list of activities *would be* for helping families feed their infants in the near future or to select “non-applicable” if they were unsure. Amongst all participants, all the activities listed in the survey were rated a mean score above 50% for helpfulness. However, the top three highest rated activities were: free prenatal lactation education (61.3%), free postpartum lactation support (59.2%), and information is available online that describes the infant formula brands that are similar and meet each infant’s unique health needs (59.7%) (Table 2). There were no differences in parental sentiment scores between WIC recipients vs. non-WIC recipients and males vs. females.

**Table 2:** Ranked Helpfulness of Proposed Resources for All Participants

	Mean	±	SD	*n*
Parents receive free lactation education during pregnancy to help prepare mothers to breastfeed	61.3	±	19.8	92
Mothers receive free lactation support by lactation consultants when their babies are born	59.2	±	20.2	97
Health insurance and Medicare pay for pasteurized donor milk	56.3	±	21.5	83
Pasteurized donor milk is sold at discounted rates	54.8	±	17.1	84
Pasteurized donor milk is free	54.0	±	20.7	83
Health insurance and Medicare pay for imported, commercially available infant formulas and not just U.S. brands	55.3	±	20.1	87
Health insurance and Medicare pay for all U.S., commercially available infant formula brands	56.8	±	18.6	85
Parents can choose to use any commercially available infant formula brand without restrictions by health insurance or Medicare	55.6	±	20.4	86
Measures are created to prevent customers from stockpiling infant formula from stores	53.6	±	19.8	91
Mothers receive free lactation support by lactation consultants to help them relactate	55.7	±	20.3	84
Online videos that help explain how to relactate are freely accessible	55.0	±	19.9	82
Information that lists where infant formula can be purchased in stores is available online	55.8	±	18.7	93
Information that describes which brands are similar and meet each baby’s unique health needs is available online	59.7	±	19.1	87
Recipes on making homemade infant formula designed by infant nutrition experts are available online	57.8	±	18.8	79

## Discussion

The 2022 infant formula shortage was an unprecedented feeding crisis that led to nationwide distress and fear as parents scrambled to find sources of nutrition for their infants [8]. This study is the first to prospectively investigate infant feeding practices in response to the 2022 infant formula shortage. Additionally, parents’ experiences and sentiments were examined to identify areas within healthcare systems and health policies that could assist in mitigating future infant feeding crisis.

In this study, 80% of the infants were combination feeders, consuming mother’s own milk and infant formula and there were no changes to the use of mother’s own breast milk in response to the shortage. A recent cross-sectional study from an online survey of U.S. caregivers of infants investigated the impact of the COVID-19 pandemic on infant feeding practices and found that at least 45% of parents engaged in behaviors to increase breastfeeding. Increased breastfeeding rates were partly motivated by concerns about the cost of infant formula, a shortage of infant formula, or because parents were running out of formula [12]. More recently, a study of electronic medical records found that breastfeeding initiation and exclusive breastfeeding rates at 1 month postpartum, and exclusive and partial breastfeeding rates at 2 months postpartum significantly increased during the 2022 infant formula shortage as compared with pre-shortage periods [20]. Our study was not designed to examine the amount of each food consumed by infants which could serve as a useful proxy for the severity of the shortage experienced by families. However, overall, the number of families that engaged in at least one deleterious feeding practice (using human milk from informal sharing, homemade infant formula, watered-down formula, and expired infant formula) in response to the shortage significantly increased from 8% to nearly half (48.5%). These data suggest that the portion of human milk consumed by infants was insufficient to fully support their diet without the use of deleterious and potentially unsafe feeding practices.

In this study, use of human milk through regulated donor milk banks significantly increased from 2% before to 28% during the shortage. These data are consistent with reports from the Human Milk Bank Association of North America (HMBANA) that experienced a high demand for donor milk during the 2022 infant formula shortage [21]. HMBANA, a non-profit organization that accredits nonprofit milk banks in the United States and Canada, produces the standards and guidelines for donated human milk in North America. Donors are screened and tested before they can donate milk, which is pasteurized and tested for communicable diseases before being entered into a milk bank’s frozen inventory and distributed. Access to banked donor milk is limited due to the number of milk banks in the U.S. (only 28) and HMBANA milk banks typically reserve their supply for infants born prematurely or whose medical needs require them to rely exclusively on human milk, and whose biological mothers cannot provide it to them. Additionally, banked donor milk is expensive ranging from $3 to $5 per ounce. The accessibility and growth of donor milk banking services are thwarted in part to a lack of federal public health policies that integrate donor milk banking or regulate its operations. For example, Brazil has 228 human milk banks and 217 collection centers throughout the country. Brazil’s thriving donor milk banking system is in part a result of its oversight by the Health Ministry that has incorporated milk banks into their health policy [22].

Due to a high demand for human milk and barriers in acquiring pasteurized banked donor milk, informal human milk sharing has become a rapidly growing infant feeding practice in the U.S [23–25]. In this study, the number of parents that used human milk from informal sharing increased from 5% before to 26% during the 2022 infant formula shortage. These rates are higher than previous reports on infant feeding practices before the 2022 infant formula shortage. For example, in a recent cross-sectional study of 2,315 individuals examining safety concerns of infant feeding trends by DiMaggio and colleagues, 8% of survey respondents reported using donor milk, of which, 36% was obtained from unregulated sources [26]. Similarly, in a cross-sectional analysis of 496 U.S. mothers, O’Sullivan et al., reported that 7% of respondents fed their child human milk from another person and 12% provided their milk to another family [27]. There are high health risks associated with feeding infants human milk from informal sharing including risks for transmission of communicable diseases such as HIV, improper collection and storage, or the risk of obtaining adulterated milk [24, 28]. Our results suggest there is a high demand for human milk regardless of its source (regulated banked vs. unregulated informal sharing). These data underscore the need for health policies that integrate donor milk banking with federal regulators and ensure access to safe banked donor milk to promote infant and child health as well as the need for an increased number of donor milk banks within the U.S.

Unsurprisingly, the use of U.S. and imported infant formulas significantly decreased during the 2022 infant formula shortage; however, the use of deleterious feeding practices including the use of watered-down infant formula significantly increased from 2% before to 29% during the 2022 infant formula shortage. These rates are consistent with a recent report by Marino et al., that investigated the impact of the COVID-19 pandemic on infant feeding practices for which 33% of families who used formula reported that they used deleterious formula-feeding practices such as diluting formula with extra water, saving leftover formula, and adding cereal to formula. Of infant formula users, 11% diluted formula with extra water. These practices were significantly more likely to be reported by families with lower compared with higher incomes [12]. Most of the respondents in our study were WIC recipients which may in part explain the higher increase in use of watered-down infant formula during the shortage. Taken together, the use of unsafe infant formula feeding practices in response to the 2022 infant formula shortage exacerbated nutrition and food insecurity in a vulnerable population and highlights the urgent need to design public programs and policies to ensure equitable and universal access to lactation support and diverse infant formula products.

During the infant formula shortage, parents relied on several resources for guidance and support related to infant feeding. 69% of respondents obtained guidance or support from healthcare providers, 68% from lactation consultants, 60% from WIC, 61% from social media, and 58% from other parents, friends, and family. These data are much lower compared with the CDC and FDA’s 2006 *Infant Feeding Practices Study II* (*IFPSII*) such that 76–82% of mothers obtained information about infant feeding practices from healthcare professionals, followed by relatives or friends, and books or videos [19]. The discrepancies between our data and IFPSII may be explained in part due to the distress in navigating the formula shortage or the differences in feeding practices between males and females [18]. In our study 65.7% of respondents were female as compared with 100% in the IFPSII.

In this study, we found differences in the utilization of resources between different populations. For example, significantly more WIC recipients reported WIC, healthcare providers and lactation consultants as resources that provided guidance or support in feeding their infants as compared with non-WIC recipients. These data may reflect the direct contact WIC recipients receive from WIC staff and associated healthcare stakeholders. Parental sex was associated with participants’ experience with resources that provided guidance or support to feed their infants. For example, compared with female parents, more males reported online resources (social media, websites by health authorities, blogs, news, and YouTube) as sources that provided guidance or support in feeding their infants.

When parents were asked to rate the helpfulness of these resources on a scale from 0 to 100%, other parents, friends, and family were rated as the most helpful (62.8%), followed by lactation consultants (62.3%), healthcare providers (59.1%), WIC (58.2%), and social media (44.6%). However, the level of helpfulness differed between females and males. For example, male parents rated healthcare providers as significantly more helpful than females (68.5% vs. 52.9%), while female parents reported higher ratings for helpfulness from social media (56.4% vs. 31.6%) and YouTube (49.4% vs. 22.4%) compared with male parents. These data suggest that outreach and education strategies on infant feeding could be most effective when communication outlets target the interest of different subsets of the parent population.

In this study, parents were asked to rate the activities that were commonly posted on social media parent groups that could be most helpful in supporting families feed their infants in the near future. The highest rated activities were lactation education during pregnancy (61.3%), followed by online websites that describe brands that are similar and meet each infant’s unique health need (59.7%), and postpartum lactation support (59.2%). These findings suggest that parents from this cohort agree that universal breastfeeding education and support are important to optimally feed infants. These findings are consistent with numerous systematic reviews that demonstrate prenatal and postpartum breastfeeding support increase the initiation, duration and exclusivity of breastfeeding (34–37).

We propose a call to action for the implementation of healthcare and government policies that prioritize lactation support and access to banked donor milk. Breastfeeding and human milk feeding are the most nutritious, safe, and reliable sources of food for infants. The American Academy of Pediatrics (AAP) and the World Health Organization (WHO) recommend exclusive breastfeeding (or feeding mother’s expressed milk) for the first six months of age or use of banked donor milk if mother’s own milk is unavailable [16, 29]. Most (84.1%) U.S. families reported ever feeding breast milk to their infants, although the rate of exclusive breastfeeding drastically decreases to 46.9% and 25.6% at 3 and 6 months of age [30]. These data suggest that while most parents aim to meet national and international health recommendations to exclusively breastfeed their infants until 6 months of age, they are unable or choose not to. The Surgeon General’s report on breastfeeding and other research have highlighted inadequate paid family and medical leave policies that prevent women from having the time and support to initiate and sustain breastfeeding; a lack of flexibility and privacy for mothers to breastfeed or pump while at work; and difficultly affording lactation services, which are not part of standard care, as explanations for the suboptimal breastfeeding rates in the U.S [31–33]. All people should be able to choose the feeding method that is best suited for their family—whether that be breastfeeding, formula feeding, or a combination—however, lower-income women often do not have the ability to make that choice, and they are more likely to rely on infant formula than breastfeeding or to start their children on formula earlier than planned [34]. Protecting infant nutrition and food security, requires systemic changes to workplace and healthcare policies that support breastfeeding and ensure equitable and reliable access to banked donor milk.

The 2022 U.S. infant formula shortage crisis highlights systemic failures and barriers to infant feeding practices that deliver optimal nutrition, safety, and food security. The U.S. infant formula supply is controlled by U.S. trade policies, regulation and tariff policies that result in a concentrated U.S. infant formula market. High tariffs on formula thwart the import of infant formula to the U.S. and federal policies that govern the manufacture and labeling of infant formula exclude them from entering the U.S. legally [5]. These geopolitics reduce the diversity of infant formula options such that only four companies: Abbott Nutrition, Nestle (Gerber), Mead Johnson (Reckitt) and Perrigo control nearly 90% of the U.S. market for infant formula [35]. This corporate monopoly has resulted in systemic failures that inequitably impacted low-income communities and nutritionally vulnerable infants. For example, WIC agencies are required to competitively bid for contracts from three U.S. infant formula manufacturers for the least expensive contract and thus propagates an oligopoly in the infant food supply chain [5]. Furthermore, infants requiring specialty formulas due to medical or other conditions had limited options for replacements of formulas during the 2022 infant formula shortage [36]. Prevention of another feeding crisis will in part depend on systemic changes to trade and regulatory policies that prioritize food security and diversification of the infant food supply.

Although this is the first report of infant feeding practices and parental experiences in response to the May 2022 infant formula shortage, there were some limitations to this study. First, the small sample size limits the generalizability of the results. With more than half of the responses from California, our findings are not representative of U.S. families that were impacted by the infant formula shortage. Notably, our survey failed to capture many responses from individuals who reside in the southern states that were hit the hardest by the shortage [17]. There is likely a high self-selection bias by targeting parents of young infants on social media who are more likely to breastfeed or combination feed. There are also several strengths of this study. First, data from this observational study was collected during the crisis rather than retrospectively, which results in a lower recall error. Second, while the sample size was small, the sample was derived from a diverse group of individuals from different demographic backgrounds including a large proportion of respondents who receive WIC benefits and may be the most impacted by the crisis. Finally, the survey collected a combination of categorical and continuous data on infant feeding, parental experiences and sentiments that could identify areas in policy and educational strategies to assist families in averting a future infant feeding crisis.

## Conclusion

This study is the first to report infant feeding practices and parental experience in response to the 2022 infant formula shortage. Responses to the shortage included practices that may pose nutritional and safety risks such as use of unregulated human milk through informal sharing and watered-down infant formula. Most parents obtained guidance or support on how to feed their infants during the shortage from a variety of resources including healthcare providers, lactation consultants, WIC, and social media. Parents rated prenatal lactation education and postpartum lactation support highest for activities that would help parents feed their infants. Our report highlights the need for less restrictive trade and regulatory policies that diversify the infant food supply. Furthermore, systemic changes to workplace and healthcare policies that integrate universal prenatal and postnatal breastfeeding support and ensure equitable and reliable access to banked donor milk are critical to protect the most vulnerable population from another feeding crisis.

## Electronic supplementary material

Below is the link to the electronic supplementary material.

Additional file 1: Infant Formula Shortage Survey. Description of data: A copy of the survey that parents were invited to complete.

Additional file 2: Distribution of Survey Respondents Across the United States. Description of data: Map depicting the distribution of survey respondents across the U.S., based on zip code provided by parents.

Additional file 3: Infant Feeding of Other Milks and Solid Food Before and During the Infant Formula Shortage. Description of data: Table depicting the frequency of feeding certain milks and solid foods 7 days before and 7 days during the infant formula shortage.

## Data Availability

The datasets used and/or analysed during the current study are available from the corresponding author on reasonable request.

## List of abbreviations

AAP: American Academy of Pediatrics
CDC: Center for Disease Control’s
HMBANA: Human Milk Bank Association of North America
IFPSII: Infant Feeding Practices Study II
U.S.: United States
WHO: Worsld Health Organization
WIC: Special Supplemental Nutrition Program for Women, Infants, and Children
