# Supplementary material for: Infant feeding practices and parental perceptions during the 2022 United States infant formula shortage crisis
Source: BMC Pediatr. 2023 Jun 24;23:320. doi: 10.1186/s12887-023-04132-9 (PMC10290398; doi:10.1186/s12887-023-04132-9)
Supplement: Supplementary file 1 — Additional file 1: Infant Formula Shortage Survey. Description of data: A copy of the survey that parents were invited to complete. [file 12887_2023_4132_MOESM1_ESM.docx]

**Additional File 3.** Infant Feeding of Other Milks and Solid Food Before and During the Infant Formula Shortage.

|  | Before |  | During |
| --- | --- | --- | --- |
| Cow milk, % (n) | 17.2% (17) |  | 25.3% (25) |
| Watered down cow milk, % (n) | 2.0% (2) |  | 12.1% (12) |
| Evaporative cow milk powder, % (n) | 1.0% (1) |  | 6.1% (6) |
| Goat milk, % (n) | 8.1% (8) |  | 10.1% (10) |
| Watered-down goat milk, % (n) | 1.0% (1) |  | 5.1% (5) |
| Plant-based milks (coconut, almond, oat, soy, rice, cashew, etc.), % (n) | 3.0% (3) |  | 6.1% (6) |
| Toddler formula, % (n) | 13.1% (13) |  | 9.1% (9) |
| 100% fruit or 100% vegetable juice, % (n) | 3.0% (3) |  | 8.1% (8) |
| Sweet drinks: juice drinks, soft drinks, soda, sweet tea, Kool Aid, etc., % (n) | 1.0% (1) |  | 1.0% (1) |
| Baby cereal, % (n) | 7.1% (7) |  | 18.2% (18) |
| Other cereals and starches: breakfast cereals, teething biscuits, crackers, breads, pasta, etc., % (n) | 1.0% (1) |  | 2.0% (2) |
| Fruit (including purees), % (n) | 6.1% (6) |  | 10.1% (10) |
| Vegetables (including purees), % (n) | 6.1% (6) |  | 8.1% (8) |
| Meats, chicken, combination dinners (including purees), % (n) | 3.0% (3) |  | 9.1% (9) |
| Fish or shellfish (including purees), % (n) | 6.1% (6) |  | 7.1% (7) |
| Eggs, % (n) | 10.1% (10) |  | 16.2% (16) |
| Nut butters or nuts, % (n) | 2.0% (2) |  | 3.0% (3) |
| Sweet foods: candy, cookies, cake, etc., % (n) | 0.0% (0) |  | 0.0% (0) |
| Other, % (n) | 0.0% (0) |  | 1.0% (1) |
