# Supplementary figures and images for: Infant feeding practices and parental perceptions during the 2022 United States infant formula shortage crisis
Source: BMC Pediatr. 2023 Jun 24;23:320. doi: 10.1186/s12887-023-04132-9 (PMC10290398; doi:10.1186/s12887-023-04132-9)

**Additional File 2.** Distribution of Survey Respondents Across the United States.


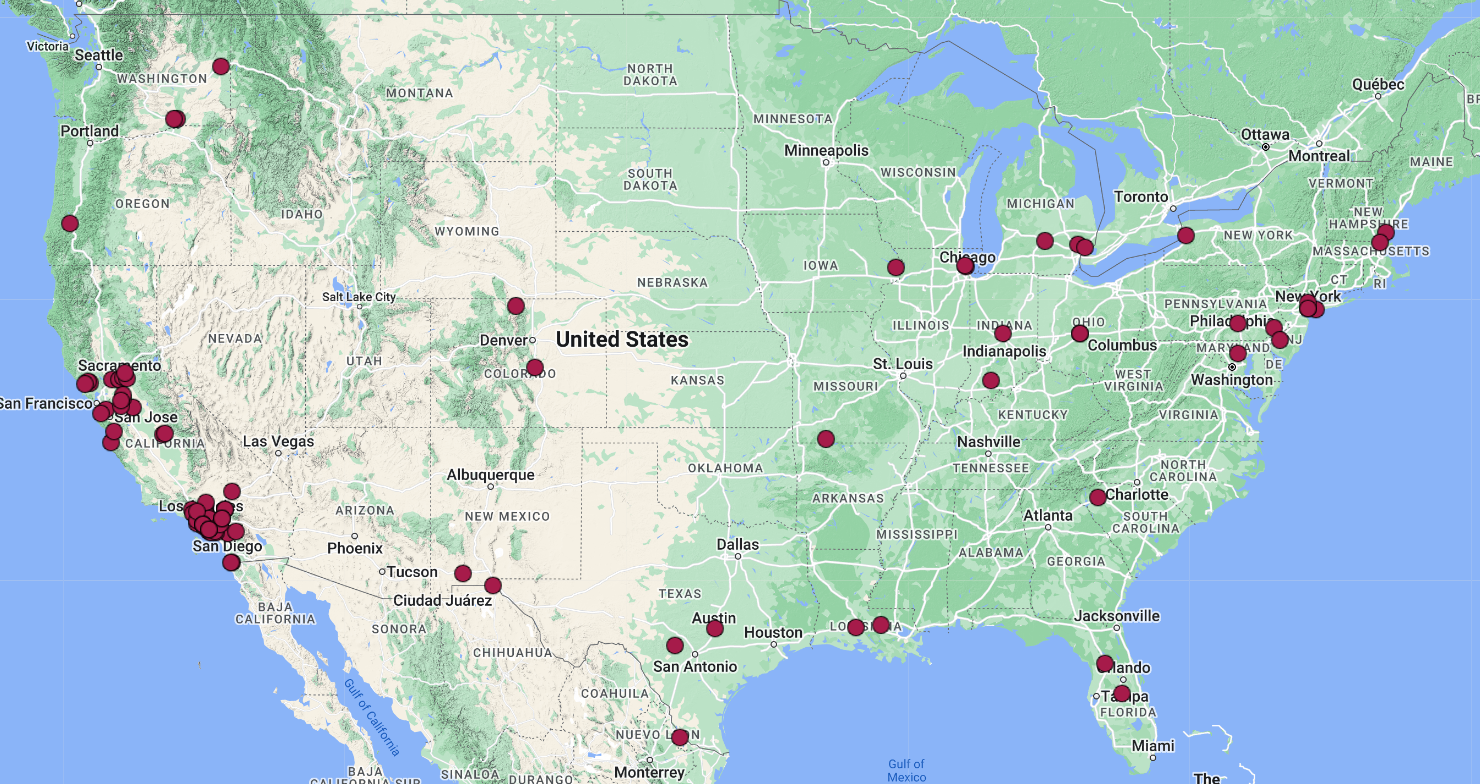

Supplement: Supplementary file 2 — Additional file 2: Distribution of Survey Respondents Across the United States. Description of data: Map depicting the distribution of survey respondents across the U.S., based on zip code provided by parents. [file 12887_2023_4132_MOESM2_ESM.docx]
