# Supplementary material for: Infant feeding practices and parental perceptions during the 2022 United States infant formula shortage crisis
Source: BMC Pediatr. 2023 Jun 24;23:320. doi: 10.1186/s12887-023-04132-9 (PMC10290398; doi:10.1186/s12887-023-04132-9)
Supplement: Supplementary file 3 — Additional file 3: Infant Feeding of Other Milks and Solid Food Before and During the Infant Formula Shortage. Description of data: Table depicting the frequency of feeding certain milks and solid foods 7 days before and 7 days during the infant formula shortage. [file 12887_2023_4132_MOESM3_ESM.docx]

**Additional File 1.** Infant Formula Shortage Survey

Start of Block: Questionnaire Instructions

1
**Infant Formula Shortage Crisis Survey**

2
**Survey Instructions**
  **Thank you for taking the time to complete this 5-minute survey!**

 The **purpose** of this survey is to learn about your experience in feeding your baby during the United States infant formula shortage that began in May 2022. We hope to use this information to create resources that could help prevent another crisis.

 Please complete this survey once if you meet all three conditions:
 1) you lived in the **United States** during the infant formula shortage in May 2022.
 2) you are the parent of a **baby (biological or not biological) who was 12 months old or younger** during the infant formula shortage in May 2022.
 3) you **experienced challenges** with feeding your baby because of the infant formula shortage in May 2022.
 
**Instructions:** Please provide your best answer to each question. Only one answer can be selected unless the instructions say to "check all that apply". If you do not know or cannot remember the answer to a question, mark "unsure". If you prefer not to answer a question, mark "decline to answer".  
 
You can select the back arrow to return to previous questions, but you will not be able to move forward until you have answered the current question. Be sure to double-check your answers before submitting, since you will not be able to go back and change your answers once you have submitted a completed questionnaire.
  
Please answer the questions honestly and to the best of your knowledge. Your answers will be kept confidential. 


You will be emailed an electronic Target gift card after completing this survey.


You may only take this survey once to receive a gift card.

 For any questions or concerns, please email Jennifer Smilowitz, jensm@ucdavis.edu

End of Block: Questionnaire Instructions

Start of Block: Survey criteria

| 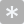 |
| --- |

3 What is your first name?

________________________________________________________________

| Page Break |  |
| --- | --- |

| 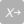 |
| --- |

4 Do you have a baby who was **12 months old or younger** during the U.S. infant formula shortage that began in May 2022?
 *[For this question, check 'yes' if you are a biological or non-biological parent, foster parent, adoptive parent, legal guardian or informal guardian]*

- Yes (1)
- No (2)
- Unsure (3)
- Decline to answer (4)

Skip To: End of Survey If Do you have a baby who was 12 months old or younger during the U.S. infant formula shortage that... != Yes

| 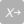 |
| --- |

5 Are you currently a resident of the United States?

- Yes (1)
- No (2)
- Unsure (3)
- Decline to answer (4)

Skip To: End of Survey If Are you currently a resident of the United States? != Yes

| 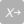 |
| --- |

6 Did you experience any challenges feeding your baby because of the U.S. infant formula shortage that began in May 2022?

- Yes (1)
- No (2)
- Unsure (3)
- Decline to answer (4)

Skip To: End of Survey If Did you experience any challenges feeding your baby because of the U.S. infant formula shortage t... != Yes

End of Block: Survey criteria

Start of Block: Demographics

7
**Demographics**

| 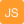 | 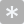 |
| --- | --- |

8 What is your **age** (in years)?

________________________________________________________________

| 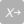 |
| --- |

9 What is your **ethnicity**?

- Hispanic or Latinx (1)
- Not Hispanic or Latinx (2)
- Unsure (3)
- Decline to answer (4)

| 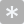 | 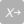 |
| --- | --- |

10 Which **race** best describes you?

- American Indian/Alaska Native (1)
- Asian (2)
- Native Hawaiian or other Pacific Islander (3)
- Black or African American (4)
- White (including Middle Eastern populations) (5)
- Other (please describe) (6) __________________________________________________
- 2 or more (please describe) (7) __________________________________________________
- Unsure (8)
- Decline to answer (9)

| Page Break |  |
| --- | --- |

| 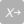 |
| --- |

11 What is the highest level of **education** you have completed?

- No schooling completed, or less than 1 year (1)
- Nursery, kindergarten, and elementary (grades K-8) (2)
- High school (grades 9-12, no degree) (3)
- High school graduate (or equivalent) (4)
- Some college (1-4 years, no degree) (5)
- Associate’s degree (including occupational or academic degrees) (6)
- Bachelor’s degree (BA, BS, etc.) (7)
- Master’s degree (MA, MS, MEng, MSW, etc.) (8)
- Professional or doctorate (MD, DDS, JD, PhD, EdD, etc) (9)
- Unsure (10)
- Decline to answer (11)

| 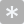 | 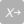 |
| --- | --- |

12 What is your **main** **occupation**?

- Agriculture / Farming (1)
- Construction (2)
- Food Preparation / Food Server / Waiter or Waitress (3)
- Health Care Worker / Doctor / Nurse (4)
- Janitor / Housekeeper (5)
- Laboratory Technician / Scientist (6)
- Manufacturing / Factory (7)
- Military (8)
- Public Safety (Police, Firefighter, Security Guard) (9)
- Repair Services / Mechanic / Plumber (10)
- Retail / Sales / Cashier (11)
- Stay at home parent (12)
- Student (13)
- Teacher / Teacher’s Aid (14)
- Warehouse / Stockroom (15)
- Work in an office environment (16)
- Not employed (17)
- Other (please describe) (18) __________________________________________________
- Unsure (19)
- Decline to answer (20)

| Page Break |  |
| --- | --- |

| 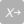 |
| --- |

13 What is your current **marital status**?

- Married/Unmarried couple (1)
- Divorced/Separated (2)
- Widowed (3)
- Never married (4)
- Decline to answer (5)

| Page Break |  |
| --- | --- |

| 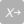 |
| --- |

14 How **many** **individuals** live in your household? *[A household includes one or several persons who live in the same home and share meals. Include yourself plus any other people who live in your household part- or full-time]*

▼ 1 (1) ... 15 (15)

| 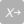 |
| --- |

15 What was your **combined household income** before taxes in the past year?

 *[Please include your income plus the income of all of the people in your household* *from all sources such as wages, salaries, Social Security or retirement benefits, help from relatives, etc.]*

- Less than $25,000 (1)
- $25,000 to $34,999 (2)
- $35,000 to $49,999 (3)
- $50,000 to $74,999 (4)
- $75,000 to $99,999 (5)
- Greater than $100,000 (6)
- Unsure (7)
- Decline to answer (8)

| 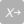 |
| --- |

16 Have **you or your baby's co-parent** received any **benefits** from the Special Supplemental Nutrition Program for Women, Infants, and Children otherwise known as WIC within the **past 12 months**?

- Yes (1)
- No (2)
- Unsure (3)
- Decline to answer (4)

| 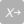 |
| --- |

17 In which state do you currently reside?

▼ Alabama (1) ... I do not reside in the United States (53)

18 What is your U.S. zip code

- Zip code: (5) __________________________________________________

| Page Break |  |
| --- | --- |

| 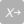 |
| --- |

19 What is your sex?

 *[We are asking about your sex to follow-up with questions about your reproductive history]*

- Female (1)
- Male (2)
- Intersex (3)
- Decline to answer (4)

End of Block: Demographics

Start of Block: Reproductive history

Display This Question:

If What is your sex?[We are asking about your sex to follow-up with questions about your reproductiv... = Female

20
**Questions about your reproductive history**

Display This Question:

If What is your sex?[We are asking about your sex to follow-up with questions about your reproductiv... = Female

| 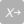 |
| --- |

21 How many times have you **ever** **been pregnant**?


*[Count* ***all****pregnancies including those that ended in live birth, stillbirth, miscarriage, abortion or a tubal, ectopic, or molar pregnancy. Include pregnancies from other relationships]*

▼ 0 (0) ... 10 (10)

Skip To: End of Block If How many times have you ever been pregnant? [Count all pregnancies including those that ended in... = 0

Display This Question:

If How many times have you ever been pregnant? [Count all pregnancies including those that ended in... != 0

And What is your sex?[We are asking about your sex to follow-up with questions about your reproductiv... = Female

| 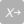 |
| --- |

22 How many times have you **delivered a live birth**?
 *[Include children from other relationships]*

▼ 0 (0) ... 10 (10)

Display This Question:

If What is your sex?[We are asking about your sex to follow-up with questions about your reproductiv... = Female

And How many times have you delivered a live birth? [Include children from other relationships] != 0

| 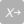 |
| --- |

23 Have you delivered a baby(s) within the **past 14 months**?

- Yes (1)
- No (2)
- Unsure (3)
- Decline to answer (4)

Skip To: End of Block If Have you delivered a baby(s) within the past 14 months? = No

Display This Question:

If What is your sex?[We are asking about your sex to follow-up with questions about your reproductiv... = Female

And Have you delivered a baby(s) within the past 14 months? = Yes

| 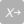 |
| --- |

24 How many babies have you delivered within the **past 14 months?**

 *[For this question we are asking about the total number of all children you have delivered in the past 12 months including multiples such as twins, triplets, etc.]*

▼ 0 (0) ... 4 (4)

Display This Question:

If What is your sex?[We are asking about your sex to follow-up with questions about your reproductiv... = Female

And How many babies have you delivered within the past 12 months? != 0

And How many babies have you delivered within the past 12 months? != 1

| 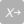 |
| --- |

25 Did you deliver more than one baby in the same labor session **within the past 14 months**?

 *[For this question we are asking if you had twins, triplets, etc.]*

- Yes (1)
- No (2)
- Unsure (3)
- Decline to answer (4)

End of Block: Reproductive history

Start of Block: Questions about the relationship to the baby

26
**Questions about how you are related to your baby**

| 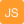 | 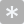 |
| --- | --- |

27 Approximately how old was your **youngest** baby when the **infant formula shortage became a problem** for you and your family?

 *[Please answer in weeks OR months only. If you have multiple babies (for example, twins or triplets, etc.) you may answer the same response for all of these babies]*

- Weeks: (1) __________________________________________________
- Months: (8) __________________________________________________

| 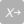 |
| --- |

28 How is your **youngest** baby related to you?

- I am a biological parent (genetically related) (1)
- I am a non-biological parent (not genetically related) (2)
- I am an adoptive parent (3)
- I am a foster parent (4)
- I am a legal guardian (5)
- I am an informal guardian (taking care of the child for a family member of friend) (6)
- Decline to answer (7)

| Page Break |  |
| --- | --- |

Display This Question:

If What is your sex?[We are asking about your sex to follow-up with questions about your reproductiv... = Female

And How is your youngest baby related to you? = I am a biological parent (genetically related)

And How many times have you delivered a live birth? [Include children from other relationships] != 0

Or How is your youngest baby related to you? = I am a non-biological parent (not genetically related)

| 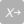 |
| --- |

29 Were you pregnant with your **youngest** baby?

- Yes (1)
- No (2)
- Decline to answer (3)

Skip To: End of Block If Were you pregnant with your youngest baby? = Yes

Display This Question:

If What is your sex?[We are asking about your sex to follow-up with questions about your reproductiv... = Female

| 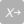 |
| --- |

30 Was your **youngest** baby born by surrogacy?

 *[Surrogacy involves using a another person to carry the pregnancy. For this question we are not asking about the biological relatedness of your baby to you or the co-parent]*

- Yes (1)
- No (2)
- Decline to answer (3)

Display This Question:

If What is your sex?[We are asking about your sex to follow-up with questions about your reproductiv... = Female

| 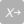 |
| --- |

31 Did you induce lactation with your **youngest** baby?

 *[New moms who did not carry their child can undergo a process of induced lactation or production of breast milk with breast stimulation through pumping and hormone therapy]*

- Yes (1)
- No (2)
- Decline to answer (3)

End of Block: Questions about the relationship to the baby

Start of Block: Lactation

Display This Question:

If What is your sex?[We are asking about your sex to follow-up with questions about your reproductiv... = Female

And Were you pregnant with your youngest baby? = Yes

Or Did you induce lactation with your youngest baby?[New moms who did not carry their child can unde... = Yes

32
**Questions about breastfeeding your baby**

Display This Question:

If What is your sex?[We are asking about your sex to follow-up with questions about your reproductiv... = Female

And Were you pregnant with your youngest baby? = Yes

Or Did you induce lactation with your youngest baby?[New moms who did not carry their child can unde... = Yes

| 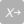 |
| --- |

33 Did your **youngest** baby ever consume**your** breast milk at the breast or by bottle?

- Yes (1)
- No (2)
- Unsure (3)
- Decline to answer (4)

Skip To: 36 If Did your youngest baby ever consume your breast milk at the breast or by bottle? = No

Display This Question:

If Did your youngest baby ever consume your breast milk at the breast or by bottle? = Yes

| 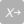 |
| --- |

34 Is your **youngest** baby still consuming **any** of **your** breast milk at the breast or by bottle?

- Yes (1)
- No (2)
- Unsure (3)
- Decline to answer (4)

Skip To: End of Block If Is your youngest baby still consuming any of your breast milk at the breast or by bottle?   = Yes

Display This Question:

If Is your youngest baby still consuming any of your breast milk at the breast or by bottle?   = No

| 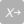 |
| --- |

35 How old was your **youngest** baby when they stopped consuming **your** breast milk at the breast or by bottle?

- Less than 1 week (1)
- Between 2-4 weeks (2)
- Between 1-2 months (3)
- Between 3-4 months (4)
- Between 5-6 months (5)
- Between 7-8 months (6)
- Between 9-10 months (7)
- Between 11-12 months (8)
- Older than 12 months (9)

Display This Question:

If Is your youngest baby still consuming any of your breast milk at the breast or by bottle?   = No

| 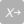 |
| --- |

36 Have you tried to relactate (restart breastfeeding) to feed your **youngest** baby **your** breast milk anytime since the start of the infant formula shortage?

 *[Relactation is when someone restarts producing breast milk after time has passed. Relactation may occur several days, weeks, months or years after lactating the first time]*

- Yes (1)
- No (2)
- Unsure (3)
- Decline to answer (4)

Display This Question:

If Have you tried to relactate (restart breastfeeding) to feed your youngest baby your breast milk a... = Yes

| 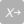 |
| --- |

37 Were you able to produce **any amount** of breast milk from relactation since the start of the infant formula shortage?

- Yes (1)
- No (2)
- Unsure (3)
- Decline to answer (4)

Skip To: 40 If Were you able to produce any amount of breast milk from relactation since the start of the infant... = No

Display This Question:

If Have you tried to relactate (restart breastfeeding) to feed your youngest baby your breast milk a... = Yes

| 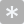 |
| --- |

38 What is the **most** amount of breast milk in **total ounces** were you able to pump in a 24-hour period from **both breasts combined** from **relactation** since the start of the infant formula shortage?

 *[Use your best guess if you are not exactly sure]*

________________________________________________________________

Display This Question:

If Have you tried to relactate (restart breastfeeding) to feed your youngest baby your breast milk a... = Yes

39 How much did **relactation** meet your **youngest** baby's daily milk needs?

*[For this question, move the marker to the percentage number that best represents how much of your baby's daily milk needs were met with relactation. 0% equals relactation did not feed your baby any breast milk, 100% equals relactation fed your baby enough breast milk to meet all of their daily milk needs and 50% is somewhere in the middle]*

|  | 0 | 10 | 20 | 30 | 40 | 50 | 60 | 70 | 80 | 90 | 100 |
| --- | --- | --- | --- | --- | --- | --- | --- | --- | --- | --- | --- |

| The percent that relactation met your baby's daily milk needs () | 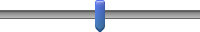 |
| --- | --- |

Display This Question:

If Have you tried to relactate (restart breastfeeding) to feed your youngest baby your breast milk a... = Yes

| 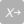 |
| --- |

40 Who has provided you with **guidance** **or support** on **relactation** to feed your **youngest** baby during the infant formula shortage?

 *[check all that apply]*

- Women Infants Children (WIC) (1)
- Healthcare providers (doctors, nurses, midwives) (2)
- Lactation consultant (3)
- Social media (Facebook, Instagram, Twitter, etc.) (4)
- Websites by health authorities (CDC, healthcare organizations, etc.) (5)
- Blogs (6)
- News (TV broadcast, online articles) (7)
- Other parents, friends, family (8)
- YouTube videos (9)
- Other (please describe) (10) __________________________________________________
- ⊗None of the above (11)

| Page Break |  |
| --- | --- |

Display This Question:

If Have you tried to relactate (restart breastfeeding) to feed your youngest baby your breast milk a... = Yes

| 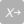 |
| --- |

41 What **challenges** or **concerns** did you have with **relactation**since the start of the infant formula shortage?

 *[check all that apply]*

- Concerned I will not produce enough milk (1)
- Concerned I will not have the time to frequently pump my milk (2)
- Concerned baby will bite (3)
- Concerned by work place will not provide enough break time required for frequent pumping (4)
- My friends or family do not support it (5)
- I do not think my milk is as good as infant formula (6)
- I prefer to have full control of my body and not nurse or pump (7)
- I did not have a good breastfeeding experience in the past (8)
- I had nipple or breast pain in the past (9)
- I do not enjoy breastfeeding (10)
- I am embarrassed about breastfeeding (11)
- My baby has a medical condition that prevents him from consuming my breast milk (12)
- I have a medical condition that prevents me from lactating (13)
- I take medicine or drugs that makes my breast milk not recommended for my baby to consume (14)
- I have a medical condition that makes my breast milk not recommended for my baby to consume (15)
- I don't know how to relactate (16)
- I don't know who can guide me to relactate (17)
- I can't find information online about how to relactate (18)
- My healthcare providers were not helpful when I asked them for guidance on relactation (19)
- Other (please describe) (20) __________________________________________________
- ⊗I did not have any challenges or concerns (21)

Display This Question:

If Is your youngest baby still consuming any of your breast milk at the breast or by bottle?   = No

And Have you tried to relactate (restart breastfeeding) to feed your youngest baby your breast milk a... = Yes

| 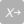 |
| --- |

42 Would you be interested in **free lactation support or guidance** to support your goal to **relactate**?

- Yes (1)
- No (2)
- Unsure (3)
- Decline to answer (4)

End of Block: Lactation

Start of Block: Feeding your baby

43
**Questions about your experience feeding your youngest baby**

| 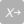 |
| --- |

44 What did your **youngest** baby typically eat over **7 days** right before the **infant formula shortage** became a **problem** for you and your family?

 *[check all that apply]*

- Breast milk (baby's mother's breast milk) (1)
- Donor human milk (from a milk bank) (2)
- Breast milk through community sharing (milk from another mother but not from a milk bank) (3)
- Infant formula (U.S. brand) (4)
- Imported infant formula (International brand) (5)
- Homemade infant formula (6)
- Watered-down infant formula (7)
- Expired infant formula (8)
- Cow milk (9)
- Watered down cow milk (10)
- Evaporative cow milk powder (11)
- Goat milk (12)
- Watered-down goat milk (13)
- Plant-based milks (coconut, almond, oat, soy, rice, cashew, etc.) (14)
- Toddler formula (15)
- 100% fruit or 100% vegetable juice (16)
- Sweet drinks: juice drinks, soft drinks, soda, sweet tea, Kool Aid, etc. (17)
- Baby cereal (18)
- Other cereals and starches: breakfast cereals, teething biscuits, crackers, breads, pasta, etc. (19)
- Fruit (including purees) (20)
- Vegetables (including purees) (21)
- Meats, chicken, combination dinners (including purees) (22)
- Fish or shellfish (including purees) (23)
- Eggs (24)
- Nut butters or nuts (25)
- Sweet foods: candy, cookies, cake, etc. (26)
- Other (please describe) (27) __________________________________________________

| Page Break |  |
| --- | --- |

| 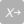 |
| --- |

45 What did your **youngest**baby typically eat over **7 days** during the the most challenging time of the **infant formula shortage** for you and your family?

 *[check all that apply]*

- Breast milk (baby's mother's breast milk) (1)
- Donor human milk (from a milk bank) (2)
- Breast milk through community sharing (milk from another mother but not from a milk bank) (3)
- Infant formula (U.S. brand) (4)
- Imported infant formula (International brand) (5)
- Homemade infant formula (6)
- Watered-down infant formula (7)
- Expired infant formula (8)
- Cow milk (9)
- Watered down cow milk (10)
- Evaporative cow milk powder (11)
- Goat milk (12)
- Watered-down goat milk (13)
- Plant-based milks (coconut, almond, oat, soy, rice, cashew, etc.) (14)
- Toddler formula (15)
- 100% fruit or 100% vegetable juice (16)
- Sweet drinks: juice drinks, soft drinks, soda, sweet tea, Kool Aid, etc. (17)
- Baby cereal (18)
- Other cereals and starches: breakfast cereals, teething biscuits, crackers, breads, pasta, etc. (19)
- Fruit (including purees) (20)
- Vegetables (including purees) (21)
- Meats, chicken, combination dinners (including purees) (22)
- Fish or shellfish (including purees) (23)
- Eggs (24)
- Nut butters or nuts (25)
- Sweet foods: candy, cookies, cake, etc. (26)
- Other (please describe) (27) __________________________________________________

| Page Break |  |
| --- | --- |

| 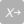 |
| --- |

46 Who has provided you with **guidance or support**in feeding your **youngest** baby during the infant formula shortage?

 *[check all that apply]*

- Women Infants Children (WIC) (1)
- Healthcare providers (doctors, nurses, midwives) (2)
- Lactation consultants (3)
- Social media (Facebook, Instagram, Twitter, etc.) (4)
- Websites by health authorities (CDC, healthcare organizations, etc.) (5)
- Blogs (6)
- News (TV broadcast, online articles) (7)
- Other parents, friends, family (8)
- YouTube videos (9)
- Other (please describe) (10) __________________________________________________
- ⊗None of the above (11)

| 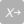 |
| --- |

47 Does your **youngest** baby require a **specialty infant formula** due to a medical or metabolic condition?

 *[Specialty infant formulas are designed to give your baby certain nutrients and assist with certain health or feeding issues. Specialty infant formulas may be recommended by a doctor for food allergies, sensitivities, gastrointestinal concerns, malabsorption, and more]*

- Yes (1)
- No (2)
- Unsure (3)
- Decline to answer (4)

| 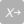 |
| --- |

48 What was the **gestational age** of your **youngest** baby at birth?

 *[For this question we are asking if your baby was born at term which is 37 weeks or later or was born premature which is less than 37 weeks]*

- Term (born 37 weeks or later) (1)
- Preterm (born before 37 weeks) (2)
- Unsure (3)
- Decline to answer (4)

| Page Break |  |
| --- | --- |

49 Rate how **helpful** the following resources **have been** with providing guidance or support to feed your **youngest** baby **in response to the infant formula shortage**.

 *[For this question, move the marker to the percentage number that best represents how helpful each resource was to you. 0% equals not helpful at all, 100% equals most helpful and 50% is somewhere in the middle. Select "not applicable" if you did not have any experiences with the listed resources]*

|  | Not Applicable |
| --- | --- |

|  | 0 | 10 | 20 | 30 | 40 | 50 | 60 | 70 | 80 | 90 | 100 |
| --- | --- | --- | --- | --- | --- | --- | --- | --- | --- | --- | --- |

| Women Infants Children (WIC) () | 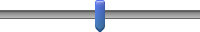 |
| --- | --- |
| Healthcare providers (doctors, nurses, midwives) () | 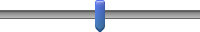 |
| Lactation consultant () | 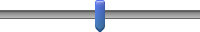 |
| Social media (Facebook, Instagram, Twitter, etc.) () | 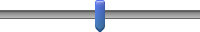 |
| Websites by health authorities (CDC, healthcare organizations, etc.) () | 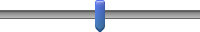 |
| Blogs () | 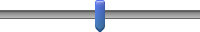 |
| News (TV broadcast, online articles) () | 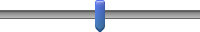 |
| Other parents, friends, family () | 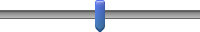 |
| YouTube videos () | 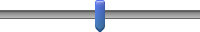 |
| Other (please describe) () | 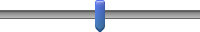 |

| Page Break |  |
| --- | --- |

50 How **helpful** do you think the following activities **would be** in **helping families feed their babies** in the near future.

 *[For this question, move the marker to the percentage number that best represents how helpful each activity could be in helping families feed their babies. 0% equals not helpful at all, 100% equals most helpful and 50% is somewhere in the middle. Select "not applicable" for activities you are unsure about]*

|  | Not Applicable |
| --- | --- |

|  | 0 | 10 | 20 | 30 | 40 | 50 | 60 | 70 | 80 | 90 | 100 |
| --- | --- | --- | --- | --- | --- | --- | --- | --- | --- | --- | --- |

| Parents receive free lactation education during pregnancy to help prepare mothers to breastfeed () | 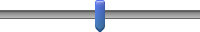 |
| --- | --- |
| Mothers receive free lactation support by lactation consultants when their babies are born () | 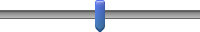 |
| Health insurance and Medicare pay for pasteurized donor milk () | 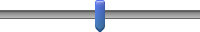 |
| Pasteurized donor milk is sold at discounted rates () | 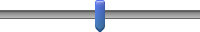 |
| Pasteurized donor milk is free () | 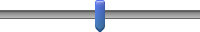 |
| Health insurance and Medicare pay for imported, commercially-available infant formulas and not just U.S. brands () | 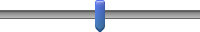 |
| Health insurance and Medicare pay for all U.S., commercially-available infant formula brands () | 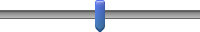 |
| Parents can choose to use any commercially-available infant formula brand without restrictions by health insurance or Medicare () | 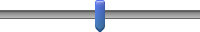 |
| Measures are created to prevent customers from stockpiling infant formula from stores () | 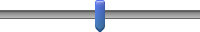 |
| Mothers receive free lactation support by lactation consultants to help them relactate () | 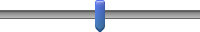 |
| Online videos that help explain how to relactate are freely accessible () | 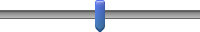 |
| Information that lists where infant formula can be purchased in stores is available online () | 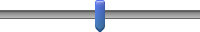 |
| Information that describes which brands are similar and meet each baby's unique health needs is available online () | 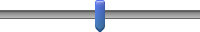 |
| Recipes on making homemade infant formula designed by infant nutrition experts are available online () | 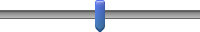 |
| Other (please describe) () | 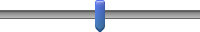 |

| Page Break |  |
| --- | --- |

51 Please share your thoughts about how you have dealt with the infant formula shortage crisis and the actions you feel should be taken by health authorities, food companies, and the government that could help you feed your baby during this crisis and prevent future crises. Write "none" if you do not wish to leave a comment.

________________________________________________________________

End of Block: Feeding your baby

Start of Block: End of Questionnaire

| 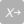 |
| --- |

52 Would you like to be contacted to learn about future research opportunities or receive information about foods for health?

 *[Check all that apply]*

- Yes to learning about future research opportunities (1)
- Yes to receiving information about foods for health (2)
- No (3)

Display This Question:

If Would you like to be contacted to learn about future research opportunities or receive informatio... = Yes to learning about future research opportunities

Or Would you like to be contacted to learn about future research opportunities or receive informatio... = Yes to receiving information about foods for health

| 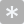 |
| --- |

53 Please write your email address so we can send you information

________________________________________________________________

54
**End of Questionnaire**
 
Please make sure your answers are complete and review them if necessary. 

 Click the **next button** once you have completed the survey and enter your email address so that we can email you a $15 Target gift card. Once you click the next button, you will not be able to go back to see your answers.

| Page Break |  |
| --- | --- |

| 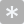 |
| --- |

55 Please enter your email address so that we can email you a $15 Target gift card.

________________________________________________________________

End of Block: End of Questionnaire
